# Role and mechanism of the AMPK pathway in waterborne Zn exposure influencing the hepatic energy metabolism of *Synechogobius hasta*

**DOI:** 10.1038/srep38716

**Published:** 2016-12-09

**Authors:** Kun Wu, Chao Huang, Xi Shi, Feng Chen, Yi-Huan Xu, Ya-Xiong Pan, Zhi Luo, Xu Liu

**Affiliations:** 1 Key Laboratory of Freshwater Animal Breeding, Ministry of Agriculture of P.R.C., Fishery College, Huazhong Agricultural University, Wuhan 430070, China; 2 Collaborative Innovation Center for Efficient and Health Production of Fisheries in Hunan Province, Changde 415000, China; 3 Panjin Guanghe Crab Co., Ltd., Panjin 124201, China

## Abstract

Previous studies have investigated the physiological responses in the liver of *Synechogobius hasta* exposed to waterborne zinc (Zn). However, at present, very little is known about the underlying molecular mechanisms of these responses. In this study, RNA sequencing (RNA-seq) was performed to analyse the differences in the hepatic transcriptomes between control and Zn-exposed *S*. *hasta*. A total of 36,339 unigenes and 1,615 bp of unigene N50 were detected. These genes were further annotated to the Nonredundant protein (NR), Nonredundant nucleotide (Nt), Swiss-Prot, Kyoto Encyclopedia of Genes and Genomes (KEGG), Clusters of Orthologous Groups (COG) and Gene Ontology (GO) databases. After 60 days of Zn exposure, 708 and 237 genes were significantly up- and down-regulated, respectively. Many differentially expressed genes (DEGs) involved in energy metabolic pathways were identified, and their expression profiles suggested increased catabolic processes and reduced biosynthetic processes. These changes indicated that waterborne Zn exposure increased the energy production and requirement, which was related to the activation of the AMPK signalling pathway. Furthermore, using the primary hepatocytes of *S*. *hasta*, we identified the role of the AMPK signalling pathway in Zn-influenced energy metabolism.

Zinc (Zn) is a ubiquitous micronutrient required for the normal growth, reproduction and development of animals, including fish. As an essential ion for more than 300 enzymes, Zn plays key roles in many aspects of cellular metabolic processes, including carbohydrate, lipid and protein metabolism. During the past several decades, extensive studies have focused on the essential roles of Zn in various biological processes and its toxic effects on many organisms^1^,^2^. Despite a considerable tolerance to high doses of Zn in some organisms^2^, excessive Zn in the aquatic environment can be toxic^1^ and has been reported to adversely impact growth, survival, reproduction, histological changes, metal bioaccumulation and the production of reactive oxygen species in fish species^3^,^4^,^5^. Accordingly, excessive Zn can pose a serious threat to the sustainable development of aquaculture. There are also studies about the effects of Zn on carbohydrate, lipid and protein metabolism^6^,^7^,^8^,^9^. However, the underlying molecular mechanism of waterborne Zn exposure in the perturbation of energy metabolism remains unclear.

In fish, the liver is one of the main sites of Zn bioaccumulation and plays a central role in energy metabolism. Protein, lipid and glucose are the major energy sources, and their balance may largely determine the energy homeostasis of the organism. Generally, disorder of energy metabolism is caused by an imbalance among energy intake from the diet, protein anabolism and catabolism, de novo fatty acid synthesis (lipogenesis)/glucose (gluconeogenesis) and fat catabolism via β-oxidation (lipolysis)/glucose breakdown (glycolysis). At present, accumulating evidence has demonstrated that cellular metabolic pathways and some kinase components play crucial roles in the regulation of energy homeostasis. Among them, AMP-activated protein kinase (AMPK) attracts wide attention because it acts as an energy sensor and regulator of energy balance at the cellular^10^ and whole-body levels^11^,^12^. AMPK is activated following a reduction of ATP levels, and more accurately, following an increase of AMP:ATP ratios. AMPK activation adjusts the ATP-generating (catabolic) and ATP-consuming (anabolic) rates^13^. In addition, recent studies have also indicated that AMPK responds to signals of its upstream kinases, such as LKB1 (liver kinase B1), CaMKK (Ca^2+^/calmodulin-dependent protein kinase kinase) and TAK1 (TGF-β-activated kinase 1)^11^,^12^.

*Synechogobius hasta*, a type of typical carnivorous fish, are widely distributed over the southern coast of Liaoning Province, China. Its commercial farming has become increasingly important in northern China because of its euryhalinity, rapid growth, good taste, and high market value^14^. However, excess hepatic lipid deposition and fatty liver occurrence led to lower survival and growth rates and reduced meat quality and harvest yields and thus posed a serious threat to the sustainable aquaculture for this fish species. Zn could adversely influence the aspects mentioned above. In humans, excessive Zn intake causes a change in the lipid profile, which forms the basis for the current upper limit of Zn intake established in the European Union^15^. Studies in our laboratory also showed that excessive Zn had effects on lipid metabolism in fish. For example, Zheng *et al*.^8^ found that waterborne Zn exposure for 4, 8, and 12 days reduced hepatic lipid deposition in *S*. *hasta*, whereas the opposite result was observed in yellow catfish subjected to Zn exposure for 56 days^9^. Furthermore, we investigated the time-course effect and mechanism of waterborne Zn exposure influencing lipid metabolism of *S*. *hasta* and found that Zn exposure reduced the hepatic lipid content by inhibiting lipogenesis and stimulating lipolysis^16^. Thus, waterborne Zn constitutes a direct link between the aquatic environment and the lipid homeostasis of fish. However, due to the lack of genomic resources, this study was limited to only a few candidate genes. A global understanding of the transcriptome profiling of *S*. *hasta* was required to further investigate the mechanism of Zn influencing physiological responses in *S*. *hasta*. To this end, transcriptome sequencing was conducted to compare the differential changes in the liver of *S*. *hasta* exposed to the control (without extra Zn addition) and 8.3 μM Zn. Furthermore, primary *S*. *hasta* hepatocytes were used to explore the potential mechanism of AMPK pathways in Zn influencing physiological responses.

## Results

### Illumina sequencing and sequence assembly

A total of 18 hepatic RNA samples, collected from 3 biological replicates of each treatment (control: C1, C2 and C3; Zn treatment: T1, T2 and T3), were subjected to RNA sequencing (RNA-seq). Approximately 340 million reads were generated, and every sample yielded 51.5 to 53.9 million clean reads (Supplementary Table 1). Of these, 97.44% of clean reads had quality scores greater than or equal to Q20 (the base quality score of 20 means an error probability of 1%, based on Phil Green’s PHRED base-calling software). Furthermore, 36,339 unigenes were detected after assembly, including 5,669 clusters and 30,670 singletons. The total length for unigenes was 33,757,047 nucleotides (nt), and the average length was 929 nt. The N50 (median length of all non-redundant sequences, with higher N50 values indicating better quality of assembly) was 1615 nt (Supplementary Table 2). The length distribution of All-Unigene is shown in Supplementary Fig. 1A. All reads have been submitted to the Sequence Read Archive at NCBI (Accession Number: SRP073412).

### Functional annotation and classification of unigenes

To verify that we annotated the unigenes, all unigene sequences were searched in the Nonredundant protein (NR), Nonredundant nucleotide (Nt), Swiss-Prot, Kyoto Encyclopedia of Genes and Genomes (KEGG), Clusters of Orthologous Groups (COG) and Gene Ontology (GO) databases. The results are shown in Supplementary Table 3, based on the cut-off e-value <0.00001. For the analysis of the protein coding region, the number of coding DNA sequences (CDSs) that mapped to the protein database was 22,694, and the number of predicted CDSs (ESTscan software) was 1,109 (Supplementary Table 3). The length distribution of CDS nucleotide sequences is shown in Supplementary Fig. 1B and C. Only unigenes mapped against CDSs were used in the subsequent analyses.

Unigenes with GO annotations accounted for 40.64% of All-Unigene (Supplementary Table 3). The terms “cellular processes”, “cell” and “cell part,” and “binding” were dominant in the categories of biological processes, cellular components and molecular functions, respectively. KEGG pathway analysis revealed that the representative pathways included metabolic pathway (11.78%), insulin signalling pathway (2.53%), AMPK (1.36%), apoptosis (1%), adipocytokine signalling pathway (0.94%) and glycolysis/gluconeogenesis (0.73%) (Table 1).

### Identification of differentially expressed genes (DEGs)

**Figure 1:**
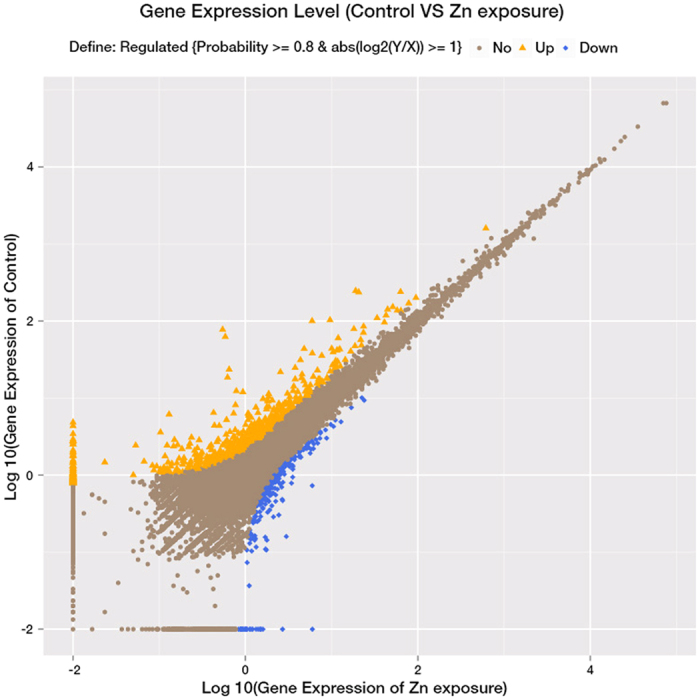
Scatter plots showing the correlation of the gene expression profiles between the control and Zn-exposed groups. X- and Y-axes present log_2_ values of gene expression. Differentially expressed genes are indicated in orange (up-regulated expression) and blue (down-regulated expression). Brown indicates genes that were not differentially expressed.

**Figure 2:**
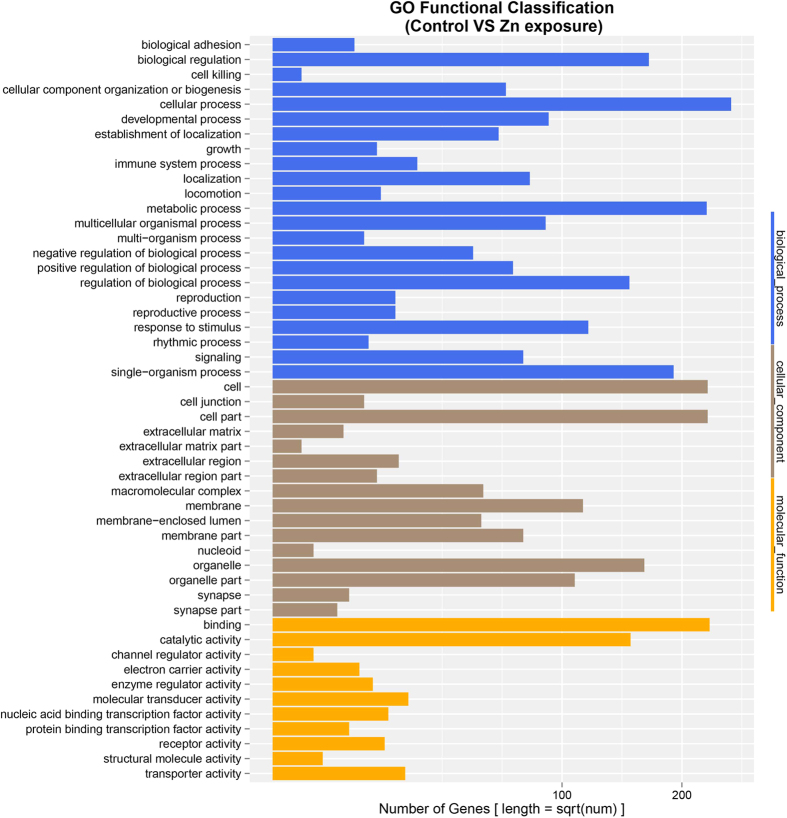
GO functional classification of differentially expressed unigenes. Unigenes were assigned to three main categories: biological process, cellular components, and molecular function.

**Figure 3:**
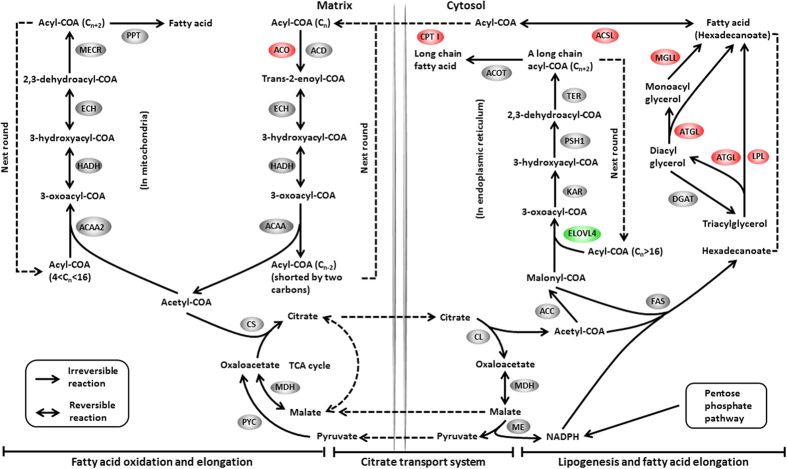
Differentially expressed genes involved in lipid metabolism. The colours of ellipses were shaded according to significance level. Red: the mRNA levels of Zn-exposed fish were significantly higher than those in the control (Probability ≥0.8, and the absolute value of log_2_(Ratio) ≥ 1). Green: the mRNA levels of Zn-exposed fish were significantly lower than those in the control (Probability ≥0.8, and the absolute value of log_2_(Ratio) ≥ 1). Grey: not DEGs.

**Figure 4:**
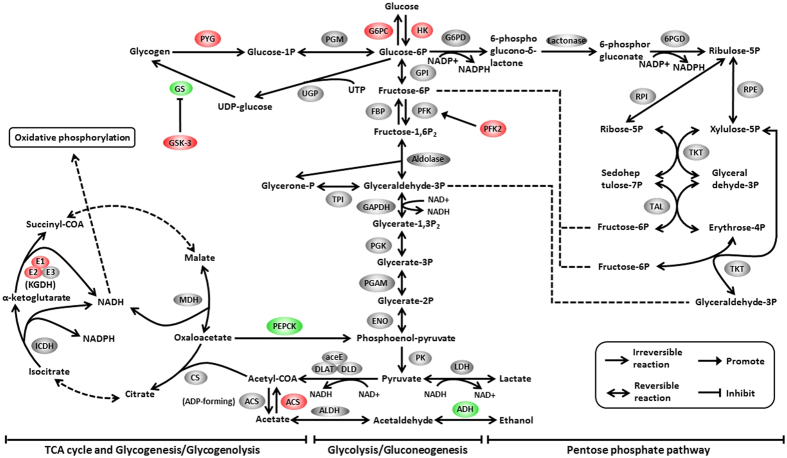
Differentially expressed genes involved in carbohydrate metabolism. The colours of ellipses were shaded according to significance level. Red: the mRNA levels of Zn-exposed fish were significantly higher than those in the control (Probability ≥0.8, and the absolute value of log_2_(Ratio) ≥ 1). Green: the mRNA levels of Zn-exposed fish were significantly lower than those in the control (Probability ≥0.8, and the absolute value of log_2_(Ratio) ≥ 1). Grey: not DEGs.

**Figure 5:**
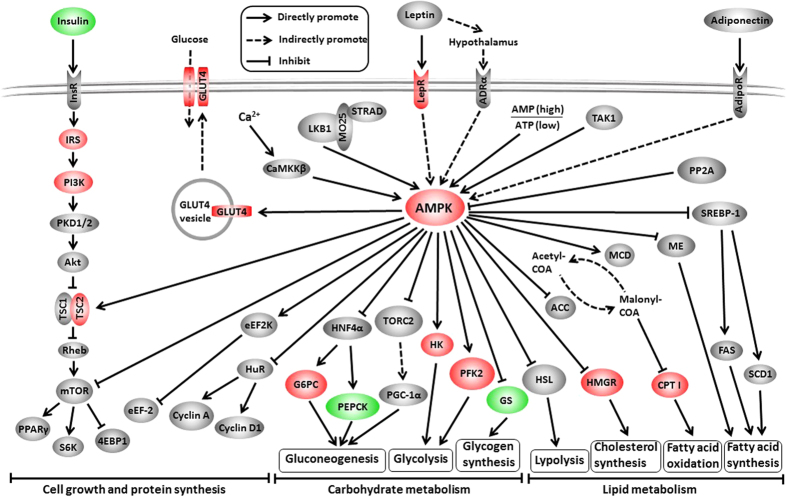
Differentially expressed genes involved in the AMPK signalling pathway. The colours of ellipses were shaded according to significance level. Red: the mRNA levels of Zn-exposed fish were significantly higher than those in the control (Probability ≥0.8, and the absolute value of log_2_(Ratio) ≥ 1). Green: the mRNA levels of Zn-exposed fish were significantly lower than those in the control (Probability ≥0.8, and the absolute value of log_2_(Ratio) ≥ 1). Grey: not DEGs.

A total of 945 unigenes were identified as DEGs between the control and Zn-exposed groups, including 708 up- and 237 down-regulated genes (Fig. 1). For the GO analysis, 255, 291 and 301 DEGs were grouped in the cellular component, molecular function and biological process categories, respectively. Within the biological processes, most of the DEGs were classified into cellular processes, followed by metabolic processes. The terms “cell” and “cell part” and “binding” were dominant in the categories of cellular components and molecular functions, respectively (Fig. 2). To characterize the functional analysis of DEGs, we performed a pathway analysis based on the KEGG database. Of the 945 DEGs, 412 had a specific KEGG pathway annotation, indicating that Zn exposure influenced different biological pathways. Furthermore, as a continuation of our previous study^16^, the present study focused on the analysis of pathways involved in energy metabolism, including lipid metabolism (Fig. 3), carbohydrate metabolism (Fig. 4) and the AMPK pathway (Fig. 5). Some key DEGs involved in oxidative phosphorylation, apoptosis, stress and repair are summarized in Supplementary Table 5. In addition, a relative RPKM (reads per kilobase per million mapped reads) value was calculated for each DEG (RPKM^gene^/RPKM^lowest^). A heatmap was constructed using log_2_ (relative RPKM value) to visualize the differential expression patterns of genes involved in lipid metabolism (Supplementary Fig. 3), carbohydrate metabolism (Supplementary Fig. 4) and the AMPK pathway (Supplementary Fig. 5). The DEGs with similar expression patterns were clearly clustered, and the abbreviations of genes are shown in the Abbreviation List of the Supplementary file.

### Validation of DEGs

To validate our RNA-seq results, 20 DEGs (15 up- and 5 down-regulated genes) were selected for validation by Q-PCR (real-time quantitative PCR). In all, except the CIDEC (cell death-inducing DFFA-like effector protein C) and HMGR (hydroxymethylglutaryl-CoA reductase) genes, the expression profiles of the other 18 genes exhibited similar trends both in RNA-seq and Q-PCR analysis (Supplementary Fig. 6). The correlation coefficient between RNA-seq and Q-PCR results was 0.892 (p < 0.001).

### Cell viability, intracellular TG (triglyceride) and ATP Content

**Figure 6:**
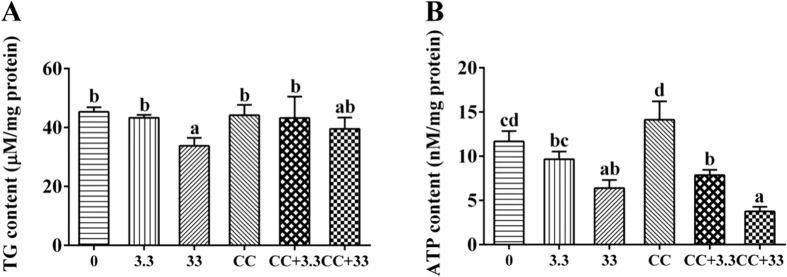
(**A**) Effect of Zn and/or Compound C (CC) on hepatocyte TG accumulation. (**B**) Effects of Zn and/or CC on ATP content in primary hepatocytes from *S*. *hasta*. (0: control; 3.3: 3.3 μM Zn; 33: 33 μM Zn; CC: 200 nM Compound C; CC + 3.3: 200 nM CC + 3.3 μM Zn; CC + 33: 200 nM CC + 33 μM Zn). Values are the means ± SEM (n = 4). Bars that share different letters indicate significant differences among groups (p < 0.05).

Compared to the control, Zn and/or the inhibitor CC (Compound C) of the AMPK pathway had no adverse effects on cell viability except in the 33 μM Zn + 200 nM CC group (Supplementary Fig. 7). Compared to the control, 3.3 μM Zn had no significant effect on intracellular TG content, but 33 μM Zn significantly reduced the TG content. Compared to the single 33 μM Zn-exposed group, CC pre-treatment and then Zn incubation resulted in a slight increase of TG, although the differences were not statistically significant (Fig. 6A). The intracellular ATP level declined with increasing Zn concentration (Fig. 6B). Compared to the single Zn exposure group, CC pre-treatment and then Zn incubation resulted in a slight reduction of intracellular ATP content, although the differences were not statistically significant.

### Enzymatic activities

**Figure 7:**
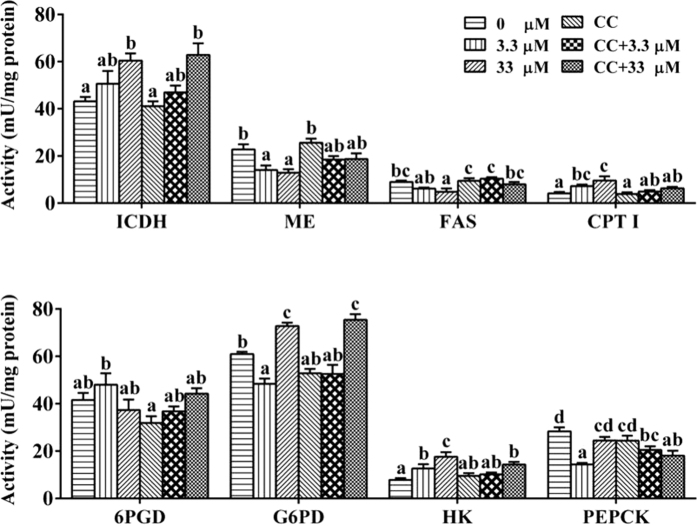
Effects of Zn and/or pathway inhibitor CC on enzymatic activities in the hepatocytes of *S*. *hasta in vitro* at 96 h. Values are expressed as the means ± SEM (n = 4). Bars that share different lowercase letters indicate significant differences among groups (p < 0.05).

Compared to the control, 3.3 μM Zn had a significant effect on the activities of ME (malic enzyme), CPT I (carnitine palmitoyl transferase I), G6PD (glucose-6-phosphate dehydrogenase), HK (hexokinase) and PEPCK (phosphoenolpyruvate carboxykinase), and 33 μM Zn significantly increased the activities of ICDH (isocitrate dehydrogenase), CPT I, G6PD and HK, and reduced the activities of ME and FAS (fatty acid synthase) (Fig. 7). For all of the tested enzymes, a single CC incubation showed no significant effects on their activities. Compared to single Zn treatment, CC pre-treatment reduced the activities of CPT I, HK and PEPCK, increased the activities of FAS in the CC + 33 μM group, and increased the activities of FAS and PEPCK in the CC + 3.3 μM group.

### mRNA expression levels of genes

**Figure 8:**
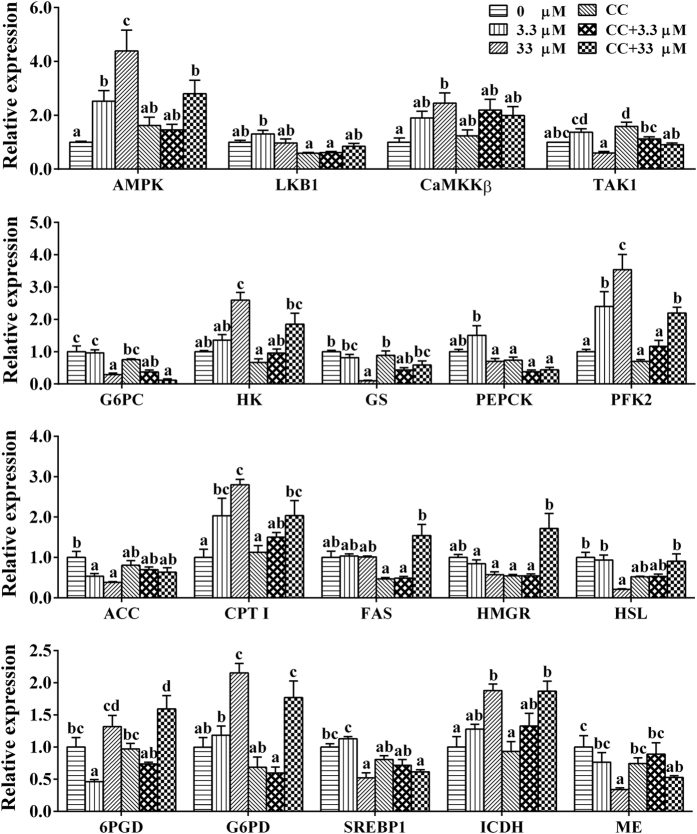
Effects of Zn and/or pathway inhibitor CC on the mRNA levels in the hepatocytes of *S*. *hasta in vitro*. The data (mean ± SEM, n = 4) were normalized to the housekeeping genes TBP and TUBA and are expressed relative to the control treatment. Bars that share different lowercase letters indicate significant differences among groups (p < 0.05).

In hepatocytes of *S*. *hasta*, compared to the control, a single CC incubation showed no significant effect on the mRNA levels of tested genes, except for TAK1 expression (Fig. 8), and 3.3 μM Zn significantly reduced the expression of ACC (acetyl-CoA carboxylase) and 6PGD (6-phosphogluconate dehydrogenase) and increased the mRNA levels of AMPK, CPT I and PFK2 (phosphofructokinase 2). The mRNA levels of AMPK, LKB1/STK11 (serine/threonine-protein kinase 11), CaMKKβ, HK, PFK2, CPT I, G6PD and ICDH were significantly higher in the 33 μM Zn-treated group compared to those in the control group. Compared to the control, 33 μM Zn exposure down-regulated the mRNA levels of G6PC (glucose-6-phosphatase), GS (glycogen synthase), ACC, HSL (hormone sensitive lipase), ME and SREBP 1 (sterol-regulator element-binding protein 1). Compared to single Zn incubation, CC pre-treatment reduced the mRNA levels of AMPK and PFK2, up-regulated the mRNA levels of GS, HMGR and HSL in the CC + 33 μM group, and reduced the mRNA levels of LKB1, G6PC, PEPCK, PFK2, G6PD and SREBP-1 in the CC + 3.3 μM group but had no significant effect on the mRNA levels of other tested genes.

## Discussion

At present, studies involving the effects of metal elements on fish often focus on oxidative damage, disease and apoptosis, and little is known about their effects and mechanisms on energy metabolism^1^,^2^,^16^. The present study provides crucial molecular insights into the mechanism of how Zn influences hepatic energy metabolism at the whole transcriptomic levels. Furthermore, using the primary hepatocytes of *S*. *hasta*, we identified a role of the AMPK signalling pathway in Zn-influenced energy metabolism in *S*. *hasta*.

In most fish species, the liver is the principal site involved in energy balance and lipid homeostasis^17^. Our recent study showed that waterborne Zn exposure reduced hepatic lipid deposition and influenced lipid metabolism of *S*. *hasta*^16^. However, due to the lack of transcriptomic information, the molecular mechanism remained unknown. In this study, using RNA-seq, we obtained many DEGs involved in lipid metabolism (Fig. 3). For example, the genes linked to fatty acid β-oxidation, such as CPT I, ACO (acyl-CoA oxidase) and ACSL (long-chain acyl-CoA synthetase), were up-regulated by Zn exposure, indicating increased lipolysis. Given that mitochondrial fatty acid β-oxidation is the major source of energy for the organism^18^, the present results likely reflected the enhancement of energy expenditure and demand after Zn exposure. Interestingly, Zheng *et al*.^9^ observed that chronic waterborne Zn exposure reduced the CPT I mRNA level in yellow catfish *Pelteobagrus fulvidraco*. This difference may be due, at least partly, to the difference of species. The present study also indicated that the mRNA levels of genes involved in glycerolipid metabolic process, such as LPL (lipoprotein lipase), ATGL (adipose triglyceride lipase) and MGLL (acylglycerol lipase), were up-regulated. LPL hydrolyses TG present in plasma lipoproteins and supplies free fatty acids (FAs) for storage or for oxidation^19^. MGLL converts monoacylglycerides to free FAs and glycerol, and ATGL is one of the main enzymes mediating TG catabolism^20^. The enhanced expression of these genes indicated the increased requirement for fatty acids, which might further promote FA oxidation. For FA elongation, ELOVL4 (elongation of very long-chain fatty acids protein 4) participates in the biosynthesis of long-chain FAs^21^. Its expression was significantly down-regulated, possibly indicating that waterborne Zn exposure inhibited long-chain FA synthesis. However, the mRNA levels of genes related to lipogenesis, such as G6PD, 6PGD, ME^22^, ACC^23^ and FAS^24^, were not significantly influenced by waterborne Zn exposure. In general, lipid homeostasis is characterized by the balance between lipolysis and lipogenesis. The present study indicated that Zn exposure mainly improved lipolysis rather than lipogenesis at the transcriptional level, indicating an increased energy expenditure.

Despite the low efficiency of carbohydrate utilization in fish, previous studies demonstrated the existence of a glucosensing system in fish and that carbohydrates are also essential for function^25^,^26^. In the present study, the changes in the mRNA levels of genes involved in gluconeogenesis did not follow a constant trend. As shown in Fig. 4, G6PC and PEPCK, two key regulatory enzymes in gluconeogenesis^27^, showed opposite changes at the transcriptional level. The present study indicated a reduction of GS expression and an increase in the PYG (glycogen phosphorylase) mRNA level after Zn exposure, indicating that Zn exposure promoted glycogen breakdown and inhibited glycogen synthesis. Of the three rate-limiting enzymes of glycolysis, the mRNA expression of HK was significantly up-regulated. Although PFK expression remained relatively stable, the mRNA level of PFK2, which activates PFK through fructose-2,6-bisphosphate production^28^, was significantly up-regulated. Thus, changes in the expression levels of these genes might indicate increased glycolysis. The tricarboxylic acid (TCA) cycle is a pivotal metabolic pathway that unifies carbohydrate, lipid and protein metabolism. KGDH (ketoglutarate dehydrogenase), ICDH and MDH (malate dehydrogenase) are critical enzymes in the TCA cycle involved in the production of NADH, which is in turn used by the oxidative phosphorylation pathway to generate adenosine triphosphate (ATP)^29^. The present study indicated that the mRNA level of KGDH was up-regulated. In addition, Zn exposure also up-regulated the expression of several key regulators involved in ATP biosynthesis, such as NADH dehydrogenase (NADH-Q, complex I), succinate dehydrogenase (SDH, complex III), cytochrome c oxidase (Cyt C Ox, complex IV) and three components (F-type subunit alpha, V-type proteolipid subunit and V-type S1 subunit) of ATP synthase (Supplementary Table 5). The increased ATP production is likely attributable to the increased metabolic expenditure for detoxification and the maintenance of homeostasis, as suggested by several researchers^6^,^8^,^9^,^22^.

The AMPK pathway is an intracellular master sensor and regulator of energy homeostasis and is activated by ATP depletion or a rise in the AMP/ATP ratio^11^,^12^. The present study indicated that Zn exposure significantly up-regulated AMPK expression (Fig. 5), suggesting the possibility of cellular ATP depletion. Similarly, Lemire *et al*.^29^ noted that Zn reduced the ATP content in hepatocytes. We also found that Zn exposure also significantly up-regulated poly (ADP-ribose) polymerase (PARP) expression (Supplementary Table 5). PARP activation is an immediate cellular response to DNA damage, and activated PARP can deplete cellular ATP and NAD^+^ contents to repair the damaged DNA^30^. In the present study, the mRNA levels of genes involved in the DNA fragmentation of apoptosis^31^,^32^, such as AIF (apoptosis-inducing factor), DFFA (DNA fragmentation factor, 45 kD, alpha subunit) and DFFB (DNA fragmentation factor, 40 kD, beta subunit), were significantly higher in the Zn-treated group than those in the control. In addition, Zn exposure changed the expression levels of calpain and caspase-3 (CASP3), key genes mediating cellular apoptosis^33^. Together, these changes suggested that excessive Zn exposure also induced DNA damage and apoptosis in the liver of *S*. *hasta*, in agreement with other reports^34^. Additionally, many pivotal genes involved in immune pathways, including NF-κB (nuclear factor NF-kappa-B), chemokine and NFAT (nuclear factor of activated T cells), were up-regulated by Zn exposure (Supplementary Table 5). NF-κB is an important redox-sensitive transcription factor that regulates gene expression involved in immune and inflammatory responses^35^. In the liver, chemokines recruit immune and non-immune cells into inflamed sites and promote wound healing^36^. The elevation of their expression levels indicated that Zn exposure influenced the immune system of *S*. *hasta*. Thus, all of the observations above indicated the Zn-induced increase of ATP depletion and the Zn-induced DNA damage and apoptosis. Taken together, it was plausible to imply that Zn exposure caused ATP depletion, which in turn activated AMPK to adjust the ATP-consumption and ATP-generation rates.

Generally, divalent metals can affect cellular calcium homeostasis and calcium signalling^37^,^38^. In the present study, waterborne Zn exposure up-regulated the expression of Ca^2+^-dependent proteins, such as calpain and calreticulin (CRT)^39^,^40^ (Supplementary Table 5). The results might reflect an elevation of cellular free Ca^2+^ concentration after waterborne Zn exposure. As we mentioned above, AMPK also responds to some protein kinases (AMPK kinase, AMPKK). Ca^2+^/calmodulin-dependent protein kinase kinase β (CaMKK β), one of AMPK’s upstream kinases^41^,^42^, senses changes in the intracellular Ca^2+^ ion concentration^43^ and has been described as the main kinase that activates AMPK in response to the elevation of intracellular Ca^2+^ levels^41^,^42^. However, the present study indicated that waterborne Zn exposure did not significantly influence the mRNA levels of CaMKKβ (Fig. 5), indicating that activation between CaMKKβ and AMPK does not always remain constant.

The present study also indicated that Zn activated the insulin signalling pathway because of the up-regulation of the mRNA expression of InsR (insulin receptor), IRS (insulin receptor substrate) and PI3K (phosphatidylinositol-4,5-bisphosphate 3-kinase). However, insulin expression was decreased. In fact, AMPK is able to activate IRS and affect insulin sensitivity by modulating the mTOR (serine/threonine-protein kinase mTOR)-S6K (p70 ribosomal S6 kinase) pathway^44^,^45^. Hence, the enhancement of insulin sensitivity may be responsible for the down-regulation of insulin expression.

Because transcriptome analysis indicated that the AMPK signalling pathway played an important role in the Zn-induced change of energy metabolism, an *in vitro* experiment using primary hepatocytes was conducted to explore its mechanism. Among several key metabolic enzymes, the activities of ME and FAS were significantly decreased while the activities of CPT I and HK increased after Zn treatment, indicating the enhancement of lipolysis, glycolysis and the reduction of lipogenesis. In the present study, 33 μM Zn incubation significantly influenced the mRNA levels of AMPK, G6PC, HK, GS, PFK2 and CPT I 6PGD, in agreement with the results of RNA-seq. As a consequence, the TG content of hepatocytes decreased in the 33 μM group (Fig. 6). The intracellular ATP level declined with increasing Zn concentration, indicating the probable activation of the AMPK pathway, which further confirmed the results of the transcriptome analysis. Meanwhile, CaMKKβ expression was up-regulated, also indicating the activation of the AMPK pathway after Zn exposure, since CaMKKβ is an important kinase upstream of AMPK^41^,^42^ (Fig. 8). Hormone-sensitive lipase (HSL), a key lipolytic enzyme, is a target gene for AMPK regulation^46^. The present study indicated that 33 μM Zn exposure significantly down-regulated HSL expression. Except for oxidative processes, the free fatty acids released by lipolysis could be re-utilized for lipogenesis. Thus, down-regulated HSL expression contributed to ensure that the rate of lipolysis did not exceed the rate of lipogenesis and thus prevented unnecessary ATP consumption induced by excessive fatty acids^14^. Similarly, previous studies demonstrated that HSL could be inhibited by AMPK activation^10^,^47^. Moreover, as shown in Figs 7 and 8, Zn incubation increased the activities and mRNA levels of G6PD and ICDH, the key regulatory enzymes involved in the production of NADPH, an essential material for lipid synthesis^21^. NADPH is also important in providing the reductive power necessary to regenerate antioxidants such as SOD and glutathione^48^. Therefore, the changes of these two NADPH-dependent enzymes likely contributed to produce large amounts of NADPH to cope with oxidative stress, as suggested by previous studies^48^,^49^. The present study indicated that, compared to single Zn treatment, CC pre-treatment reduced the activities of CPT I, HK and PEPCK and the mRNA levels of AMPK and PFK2. CC pre-treatment increased FAS activity and the mRNA levels of GS, HMGR and HSL in the CC + 33 μM group and increased the activities of FAS and PEPCK and down-regulated the mRNA levels of LKB1, G6PC, PEPCK, PFK2 and G6PD in the CC + 3.3 μM group. These findings indicated that these genes were potential targets of AMPK, in agreement with the report by Kahn *et al*.^13^. Compared to single Zn groups, CC pre-treatment significantly reduced the mRNA levels of AMPK in the CC + 33 μM Zn group. Thus, our study suggested that CC addition caused the reversion of the Zn-induced change on AMPK expression in the higher Zn group and accordingly affected the expression of downstream genes. In the present study, compared to the single Zn exposure group, CC pre-treatment and then Zn incubation resulted in a reduction of ATP content, although the differences were not statistically significant (Fig. 6). Given the close correlation between AMPK and the ATP level, the result might indicate that AMPK activation contributed to maintain a relatively adequate ATP level. The present study indicated that the 33 μM Zn + 200 nM CC group exhibited reduced cell viability. Studies have noted that intracellular ATP depletion induces cell death^50^. Taken together, the present study indicated that AMPK signals constitute a pivotal link between the ATP level and energy metabolism at the transcriptional level and that AMPK activation may be required for an ATP-conserving mechanism in response to Zn exposure.

In conclusion, this study evaluated the effects of waterborne Zn exposure on hepatic metabolism in *S*. *hasta* at the transcriptomic level and found that Zn exposure promoted catabolic processes and inhibited biosynthetic processes. AMPK played an important role in the Zn-induced changes in these genes’ expression levels and pathways. Furthermore, *in vitro* evidence suggested that the changes of energy metabolism compensated for the enhancement of energy demands, which might be attributable to the ATP-conserving mechanism of the AMPK signalling pathway in response to Zn exposure.

## Materials and Methods

The study consisted of two experiments. In Exp. 1, RNA-seq technology was used to explore the effects and mechanisms of waterborne Zn exposure on signalling pathways at the genome-wide level. Based on the results of Exp. 1, Exp. 2 was conducted to investigate the potential mechanism of AMPK pathways in Zn-influenced hepatic physiological changes of *S*. *hasta*. We assured that the experiments performed on animals and cells followed the ethical guidelines of Huazhong Agricultural University and confirmed that all experimental protocols were approved by Huazhong Agricultural University.

### Experiment 1: Transcriptome analysis

#### Fish and Zn exposure

*S*. *hasta* were obtained from a local marine water pond (Panjin, China), and the culture experiment was performed following the procedures described in our recent study^16^. Briefly, the fish were transferred to indoor cylindrical fibreglass tanks (300 L water volume) for two weeks of acclimation. After acclimation, 144 uniform-sized fish (initial mean weight: 11.3 ± 0.3 g, mean ± SEM) were randomly assigned to 6 fibreglass tanks with 24 fish per tank (200 L in water volume). They were exposed to two nominal Zn concentrations of zero (control, without extra Zn addition) and 8.3 μM (0.75% of the 96 h 50% lethal concentration [LC_50_] of Zn for *S*. *hasta*, Zheng *et al*.^8^), respectively, with triplicates for each concentration. Zn was added as ZnSO_4_·7H_2_O (AR, Shanghai Sinopharm Group Corporation, Shanghai, China) and was dissolved in distilled water for stock concentrations. Individual test solutions during the experiment were obtained by adding the appropriate volume of the primary stock to the dilution. The Zn concentrations in the test tanks were monitored twice every week by inductively coupled plasma atomic emission spectrometry (ICP-AES). The measured Zn concentrations for the two Zn treatments were 0.08 ± 0.03 and 8.4 ± 0.1 μM, respectively.

The experiment was conducted in a semi-static aquarium system at ambient temperature with a natural photoperiod provided with continuous aeration to maintain the dissolved oxygen level near saturation. All fish were fed 6% of their biomass daily (two meals per day) with minced trash fish. After 15 min, the uneaten food was removed from the tanks. The amount of food consumed by the fish in each tank was recorded daily, and the Zn concentrations used here did not affect the feeding rate. Meanwhile, to ensure good water quality and to maintain waterborne Zn levels, water was renewed twice daily. Water quality parameters were monitored twice a week in the morning. The parameters were as follows: water temperature 23.7 ± 3.2 °C; pH 8.3 ± 0.2; dissolved oxygen 0.22 ± 0.01 mM; salinity 18.9 ± 0.4%; total hardness 78.6 ± 1.5 mM and total alkalinity 6.6 ± 0.3 mM. The experiment continued for 60 days.

#### Sampling and RNA isolation

At the end of the 60-day period, the fish were starved for 24 h before sampling. After euthanizing with MS-222 (tricaine methanesulfonate, 0.38 mM), 3 fish were randomly selected from each tank (Note: the remaining fish per tank were used for histochemical observation, the determination of enzymatic activities and mRNA analysis, as described in our previous study ref. 16). Liver samples were excised and immediately frozen in liquid nitrogen. Total RNA was extracted using TRIzol reagent (Invitrogen, Carlsbad, CA, USA) according to the manufacturer’s protocol. The RNA quality and quantity were measured using a NanoDrop 2000 (Thermo Scientific, Wilmington, DE, USA) and an Agilent 2100 Bioanalyzer (Agilent Technologies, Palo Alto, CA, USA). All of the samples were standardized to 500 ng/μL, and 3 RNA samples (equal volumes) from the same tank (the same biological replicate) were combined into one pool for transcriptome analysis. There were three replicate tanks (n = 3 biological replicates) for transcriptome analysis for each treatment.

#### Library preparation and Illumina sequencing

The transcriptome library construction and sequencing were performed at the Beijing Genome Institute (BGI, Shenzhen, China). RNA samples were digested by DNase I, and magnetic beads with Oligo (dT) were used to isolate mRNA (Dynabeads mRNA Purification Kit, Invitrogen, CA, US). The mRNA was sheared to yield short fragments with Fragment Buffer (Ambion^®^, ThermoFisher Scientific, MA, US), and the fragments were used as templates for cDNA synthesis. N6 primer, First Strand Master Mix and Super Script II reverse transcription (Invitrogen, CA, US) were mixed together and incubated at 25 °C for 10 min, 42 °C for 30 min and 70 °C for 15 min to synthesize the first strand cDNA. Then, the Second Strand Master Mix (Invitrogen) was added to the solution, which was then incubated at 16 °C for 2 h to synthesize the second strand cDNA. The cDNA fragments were purified with a QIAquick PCR Purification Kit (QIAGEN, Germany). The purified cDNA fragments were resolved with EB buffer and used for end reparation and single nucleotide A (adenine) addition. They were then connected to adapters. Afterwards, the suitable fragments (300–350 bp in size) were selected for the PCR amplification. The amplified products were purified to create cDNA libraries. The Agilent 2100 Bioanalyzer and the ABI StepOnePlus Real-Time PCR System were used to quantify and qualify the sample libraries. Finally, the qualified libraries were amplified on a cBot to generate the cluster on the flowcell (TruSeq PE Cluster Kit V3-cBot-HS, Illumina, San Diego, CA, USA). The amplified flowcell was sequenced on a HiSeq 2000 System (TruSeq SBS KIT-HS V3, Illumina). In total, six libraries were generated in the present study and were run in two lanes on the Illumina platform.

#### Assembly and functional annotation

Following sequencing, the raw image data were obtained and transformed by base calling for sequence data (90-bp raw paired-end reads). After filtering adaptor sequences and low-quality bases from the reads, we used the clean reads (above 4 G per sample) for bioinformatics analysis. De novo transcriptome assembly was achieved using Trinity^51^. The resulting sequences were considered to be unigenes. To annotate the transcriptome, we performed the BLASTx alignment (e-value <10^−5^) between unigenes and protein databases, including NR, Nt, Swiss-Prot, KEGG and COG. Genes were identified according to the best hits against known sequences. GO functional annotation was accomplished with Blast2GO software^52^, and further gene classifications were performed using the WEGO program^53^.

#### DEGs and Q-PCR validation

The gene expression levels were calculated using the RPKM method^54^,^55^. DEGs were screened using Noiseq Method55 with a threshold of 0.8 (diverge probability ≥0.8). For pathway and GO enrichment analysis, all DEGs were mapped to terms in GO and the KEGG database.

Twenty candidate genes involved in lipid and carbohydrate metabolism, the AMPK pathway and signal transduction were selected for real-time quantitative PCR (Q-PCR) validation. Total RNA from control and Zn-exposed groups (n = 3 replicate tanks, two fish were sampled for each tank, and samples were not pooled) was extracted as described above. Total RNA was quantified spectrophotometrically, and the integrity was assessed by agarose gel electrophoresis. First-strand cDNA was synthesized using a PrimeScript^TM^ RT reagent Kit with gDNA Eraser (TaKaRa, Dalian, China). Q-PCR was performed in a quantitative thermal cycler (MyiQ™ 2 TwoColor Quantitative PCR Detection System, BIO-RAD, CA, USA) with a 20-μL reaction volume containing 10 μL SYBR Premix Ex Taq™ II (TaKaRa, Dalian, China), 1 μL of diluted cDNA (10-fold), 10 mM each of forward and reverse primers (0.4 μL each) and 8.2 μL double-distilled H_2_O. Primers are given in Supplementary Table 4. The Q-PCR parameters consisted of initial denaturation at 95 °C for 30 s, followed by 40 cycles at 95 °C for 5 s, 57 °C for 30 s and 72 °C for 30 s. All reactions were performed in duplicate, and each reaction was confirmed to contain a single product of the correct size by agarose gel electrophoresis. A non-template control and dissociation curve were performed to ensure that only one PCR product was amplified and that stock solutions were not contaminated. Standard curves were constructed for each gene using serial dilutions of stock cDNA. The amplification efficiencies of all genes were approximately equal and ranged from 97% to 102%. A set of seven housekeeping genes, including β-actin, GAPDH (glyceraldehyde-3-phosphate dehydrogenase), RPL7 (ribosomal protein L7), 18 S rRNA, HPRT (hypoxanthine-guanine phosphoribosyl transferase), UBCE (ubiquitin-conjugating enzyme) and TUBA (tubulin alpha chain) were selected from the literature^56^ to test their stability of mRNA expression. The mRNA expression levels of β-actin and RPL7 were the most stable under the experimental conditions, as suggested by geNorm software^56^. Thus, the expression levels of each tested gene were normalized to the geometric mean of the best combination of β-actin and RPL7. The fold changes in relative expression to the control were calculated using the 2^−ΔΔCt^ method^57^.

### Experiment 2: Treatments *in vitro*

#### Hepatocyte culture and treatments

Hepatocytes were isolated from juvenile *S*. *hasta* according to our recent study^58^, and the cells were counted in a haemocytometer. Trypan blue exclusion was utilized to evaluate cell viability, and only those cultures with more than 95% cell viability were accepted for the subsequent experiment. The freshly isolated hepatocytes were seeded at a density of 1 × 10^6^ cells/mL onto 25 cm^2^ flasks and kept at 28 °C in a CO_2_ incubator (0.5% CO_2_). For each culture, a pool of cells from four fish was used.

For the Zn-exposed experiment, hepatocytes of *S*. *hasta* were incubated with Zn and/or CC (Dorsomorphin, Selleck S7306, Selleck Chemicals, Houston, TX, USA), a widely used AMPK inhibitor. Here, six groups were designed as follows: control, 3.3 μM Zn (0.75% of the 96-h IC_50_ of Zn for *S*. *hasta* hepatocytes. Here, IC_50_ represents concentration resulting in 50% inhibition of cell growth), 33 μM Zn (7.5% of the 96 h IC_50_), 200 nM CC, 3.3 μM Zn + 200 nM CC and 33 μM Zn + 200 nM CC. The groups containing CC were pre-treated with CC for 1 h prior to the addition of Zn. The 96 h IC_50_ (436.78 μM) of Zn for *S*. *hasta* hepatocytes was obtained from our preliminary experiment. The concentration of inhibitor was selected according to our preliminary experiment and according to previous *in vitro* studies^59^,^60^. The hepatocytes were maintained in M199 medium (M199, Gibco/Invitrogen, UK) containing 1 mmol/L L-glutamine, 5% (v/v) foetal bovine serum (FBS, Gibco/Invitrogen, UK), penicillin (100 IU/mL) and streptomycin (100 μg/mL). Each treatment was performed in quadruplicate.

#### Cell viability, TG and ATP content

The 3-(4,5-dimethylthiazol-2-yl)-2,5-diphenyltetrazolium bromide (MTT) assay was used to test cell viability according to our previous study^59^. The intracellular TG content was determined by glycerol-3-phosphate oxidase p-aminophenol (GPO-PAP) methods using a commercial kit from Nanjing Jian Cheng Bioengineering Institute (Nanjing, China). ATP was measured using an ATP Assay Kit (Beyotime, Haimen, China).

#### Analysis of enzymatic activity and Q-PCR

For assays of the activities of FAS, G6PD, 6PGD, ICDH and ME, the cells were homogenized by sonication in extraction buffer (0.02 M Tris-HCl, 0.25 M sucrose, 2 mM EDTA, 0.1 M sodium fluoride, 0.5 mM phenyl methyl sulphonyl fluoride, and 0.01 M β-mercaptoethanol, pH 7.4). FAS activity was determined by the method of Chakrabarty and Leveille^61^, G6PD activity following the method of Barroso *et al*.^62^, and 6PGD activity according to the method of Hisar *et al*.^63^. The ICDH and ME activities were measured according to Pierron *et al*.^64^. CPT I activity was measured according to a modified protocol from Morash *et al*.^65^. Briefly, the cells were homogenized by sonication in extraction buffer (250 mM sucrose, 1 mM EDTA, 20 mM HEPES, and 0.5% bovine serum albumin [BSA], pH 7.4). The reaction mixture contained 0.1 mM 5,5′-dithiobis (2-nitrobenzoic acid) (DTNB), 5 mM L-carnitine and 0.1 mM palmitoyl-CoA. CPT I activity was measured in the forward direction (formation of palmitoylcarnitine) by monitoring the initial rate of CoA-SH release with DTNB at 412 nm. The HK and PEPCK activities were measured according to Polakof *et al*.^26^. The protein content was measured following the method of Bradford^66^, with BSA used as the standard. One unit of enzyme activity was defined as 1 μM of substrate converted to product per minute at 28 °C and was expressed as mU mg^−1^ soluble protein.

Q-PCR for the *in vitro* experiment was performed using the protocol described above. Among a set of eight housekeeping genes (β-actin, GAPDH, RPL7, 18 S rRNA, HPRT, TBP, TUBA and UBCE), the mRNA expression of TBP and TUBA proved to be the most stable across the experimental conditions *in vitro* according to geNorm software. Primers are given in Supplementary Table 4.

#### Statistical analysis

Statistical analyses were performed with SPSS 19.0 software (SPSS, Michigan Avenue, Chicago, IL, USA). The results are presented as the means ± SEM (standard errors of means). Prior to statistical analysis, all data were evaluated for normality using the Kolmogorov-Smirnov test. Bartlett’s test was performed for testing the homogeneity of variances. Then, the data were subjected to one-way ANOVA followed by Turkey’s multiple range tests. Significant differences were established at p < 0.05.

## Additional Information

**How to cite this article**: Wu, K. *et al*. Role and mechanism of the AMPK pathway in waterborne Zn exposure influencing the hepatic energy metabolism of *Synechogobius hasta*. *Sci. Rep.* **6**, 38716; doi: 10.1038/srep38716 (2016).

**Publisher's note:** Springer Nature remains neutral with regard to jurisdictional claims in published maps and institutional affiliations.

## Supplementary Material

Supplementary Tables and Figures

## Figures and Tables

**Table 1:** Annotation and DEGs of pathways (results were determined using KEGG).

Pathway	All-Unigenes with pathway annotation (16937)		DEGs with pathway annotation (412)	
	Number	Percentage	Number	Percentage
Metabolic pathways	1996	11.78%	62	15.05%
Insulin signalling pathway	398	2.35%	27	6.55%
AMPK signalling pathway	230	1.36%	18	4.37%
Apoptosis	169	1%	8	1.94%
Adipocytokine signalling pathway	159	0.94%	12	2.91%
Starch and sucrose metabolism	138	0.81%	11	2.67%
Glycolysis/gluconeogenesis	124	0.73	9	2.18%
Steroid biosynthesis	39	0.23%	6	1.46%
