# Supplementary material for: Role and mechanism of the AMPK pathway in waterborne Zn exposure influencing the hepatic energy metabolism of Synechogobius hasta
Source: Sci Rep. 2016 Dec 9;6:38716. doi: 10.1038/srep38716 (PMC5146659; doi:10.1038/srep38716)
Supplement: Supplementary Tables and Figures [file srep38716-s1.doc]

**Role and mechanism of AMPK pathway in waterborne Zn exposure influencing** **hepatic energy metabolism of *Synechogobius hasta***

Kun Wu^1^, Chao Huang^1^, Xi Shi^1^, Feng Chen^1^, Yi-Huan Xu^1^, Ya-Xiong Pan^1^, Zhi Luo^1,2^,*, Xu Liu^3^

^1^Key Laboratory of Freshwater Animal Breeding, Ministry of Agriculture of P.R.C., Fishery College, Huazhong Agricultural University, Wuhan 430070, China. ^2^Collaborative Innovation Center for Efficient and Health Production of Fisheries in Hunan Province, Changde 415000, China.

^3^Panjin Guanghe Crab Co., Ltd, Panjin 124201, China

***Corresponding author. Prof. Zhi Luo, Tel.: +86-27-8728-2113; Fax: +86-27-8728-2114; Email address: luozhi99@mail.hzau.edu.cn; luozhi99@aliyun.com (Z. Luo).

**Abbreviation List:**

4EBP 1, eukaryotic translation initiation factor 4E binding protein 1; 6PGD, 6-phosphogluconate dehydrogenase.

ACAA, acetyl-CoA acyltransferase; ACC, acetyl-CoA carboxylase; ACD, acyl-CoA dehydrogenase; aceE, pyruvate dehydrogenase E1 component; ACO, acyl-CoA oxidase; ACOT, acyl-coenzyme A thioesterase; ACS, acetyl-CoA synthetase; ACSL, long-chain acyl-CoA synthetase; ADH, alcohol dehydrogenase; AdipoR, adiponectin receptor; ADRα, adrenergic receptor alpha; AIF, apoptosis-inducing factor; Akt, RAC serine/threonine-protein kinase; ALDH, aldehyde dehydrogenase; AMPK, AMP-activated protein kinase; ATGL, [adipose triglyceride lipase](https://en.wikipedia.org/wiki/Adipose_triglyceride_lipase).

CAMKKβ, calcium/calmodulin-dependent protein kinase kinase β; CASP 3, caspase-3; CL, citrate lyase; CIDEC, Cell death-inducing DFFA-like effector protein C; CPT I, carnitine palmitoyl transferases I; CRT, calreticulin; CS, citrate synthase; Cyt C Ox, Cytochrome c oxidase.

DDIT4, DNA-damage-inducible transcript 4; DFFA, DNA fragmentation factor, 45 kD, alpha subunit; DFFB, DNA fragmentation factor, 40 kD, beta subunit; DGAT, diacylglycerol acyltransferase; DLAT, pyruvate dehydrogenase E2 component; DLD, dihydrolipoamide dehydrogenase.

ECH, enoyl-CoA hydratase; eEF2, elongation factor 2; eEF2K, elongation factor 2 kinase; ELOVL, elongation of very long chain fatty acids protein; ENO, enolase.

FAS, fatty acid synthase; FBP, fructose-1,6-bisphosphatase.

G6PC, glucose-6-phosphatase; G6PD, glucose-6-phosphate dehydrogenase; GAPDH, glyceraldehyde-3-phosphate dehydrogenase; GLUT4, solute carrier family 2 (facilitated glucose transporter), member 4; GPI, glucosephosphate isomerase; GS, glycogen synthase; GSK-3, glycogen synthase kinase 3.

HADH, 3-hydroxyacyl-CoA dehydrogenase; HK, hexokinase; HMGR, hydroxymethylglutaryl-CoA reductase; HNF4α, hepatocyte nuclear factor 4-alpha; HPRT, hypoxanthine-guanine phosphoribosyl transferase; HSL, hormone sensitive lipase; HuR, ELAV-like protein 1.

ICDH, isocitrate dehydrogenase; ILF2, interleukin enhancer-binding factor 2; InsR, insulin receptor; IRS, insulin receptor substrate.

KAR, very-long-chain 3-oxoacyl-CoA reductase; KGDH, ketoglutarate dehydrogenase.

LDH, lactate dehydrogenase; LepR, leptin receptor; LKB1/STK11, serine/threonine-protein kinase 11; LPL, lipoprotein lipase.

MCD, malonyl-CoA decarboxylase; MDH, malate dehydrogenase; ME, malic enzyme; MECR, mitochondrial trans-2-enoyl-CoA reductase; MGLL, acylglycerol lipase; MO25, calcium binding protein 39; MS-222, tricaine methanesulfonate; mTOR, serine/threonine-protein kinase mTOR.

NADH-Q, NADH dehydrogenase; NAMPT, nicotinamide phosphoribosyltransferase; NFAT, nuclear factor of activated T-cells; NF-κB, nuclear factor NF-kappa-B.

PARP, poly (ADP-ribose) polymerase; PEPCK, phosphoenolpyruvate carboxykinase; PERK, PKR-like ER kinase; PFK, phosphofructokinase; PGAM, phosphoglyceromutase; PGC-1α, alpha peroxisome proliferator-activated receptor gamma coactivator 1-alpha; PGK, phosphoglycerate kinase; PGM, phosphoglucomutase; PI3K, phosphatidylinositol-4,5-bisphosphate 3-kinase; PK, pyruvate kinase; PKD, 3-phosphoinositide dependent protein kinase; PP2A, serine/threonine-protein phosphatase 2A; PPAR, peroxisome proliferators-activated receptor; PPT, palmitoyl-protein thioesterase; PSH1, very-long-chain (3R)-3-hydroxyacyl-CoA dehydratase; PYC, pyruvate carboxylase; PYG, glycogen phosphorylase.

Rheb, Ras homolog enriched in brain; RPE, ribulose-5-phosphate epimerase; RPI, ribulose-5-phosphate isomerase; RPL7, ribosomal protein L7.

S6K, p70 ribosomal S6 kinase; SCD1, stearoyl-CoA desaturase 1; SDH, succinate dehydrogenase; SOCS2, suppressor of cytokine signaling 2; SREBP 1, sterol-regulator element-binding protein 1; STRAD, STE20-related kinase adapter protein.

TAK1, mitogen-activated protein kinase kinase kinase 7; TAL, transaldolase; TAP2, transporter-associated with antigen processing 2; TBP, TATA-box-binding protein; TER, very-long-chain enoyl-CoA reductase; TKT, transketolase; TORC2, CREB-regulated transcription coactivator 2; TPI, triose phosphofructokinase; TSC, tuberous sclerosis; TUBA, tubulin alpha chain.

UBCE, ubiquitin-conjugating enzyme; UGP, UDP-glucose pyrophosphorylase.

**Supplementary Table 1 Summary of output statistics by Illumina sequencing**

| Samples | Total raw reads | Total clean reads | Total clean Nucleotides | %Bases ≥ Q20 | N percentage | GC percentage |
| --- | --- | --- | --- | --- | --- | --- |
| C1 | 55,120,344 | 51,501,262 | 4,635,113,580 | 97.43% | 0.00% | 49.53% |
| C2 | 56,228,950 | 52,612,456 | 4,735,121,040 | 97.47% | 0.00% | 49.26% |
| C3 | 56,477,578 | 52,717,092 | 4,744,538,280 | 97.41% | 0.00% | 49.44% |
| T1 | 56,957,950 | 53,234,074 | 4,791,066,660 | 97.43% | 0.00% | 49.24% |
| T2 | 56,957,950 | 53,868,544 | 4,848,168,960 | 97.44% | 0.00% | 49.33% |
| T3 | 57,688,480 | 53,868,544 | 4,848,168,960 | 97.44% | 0.00% | 49.59% |

C1, C2 and C3 represent 3 biological replicates of control group; T1, T2 and T3 represent 3 biological replicates of Zn-treated group. Total Reads and Total Nucleotides (nt) are actually clean reads and clean nucleotides; Q20 indicates a base call accuracy of 99% (1:100 probability of incorrect base call); N percentage is proportion of unknown nucleotides in clean reads; GC percentage is proportion of guanidine and cytosine nucleotides among total nucleotides.

**Supplementary Table 2 Statistics of assembly quality**

|  | Sample | | | Total number | Total length (nt) | Mean length (nt) | N50 (nt) | Total consensus Sequences | Distinct Clusters | Distinct Singletons |
| --- | --- | --- | --- | --- | --- | --- | --- | --- | --- | --- |
| Contig | | C1 | 64,337 | | 25,054,228 | 389 | 727 |  |  |  |
|  | | C2 | 64,361 | | 25,112,436 | 390 | 727 |  |  |  |
|  | | C3 | 66,264 | | 26,105,381 | 394 | 741 |  |  |  |
|  | | T1 | 66,650 | | 26,688,119 | 400 | 765 |  |  |  |
|  | | T2 | 67,021 | | 26,966,556 | 402 | 777 |  |  |  |
|  | | T3 | 64,955 | | 26,264,297 | 404 | 784 |  |  |  |
| Unigene | | C1 | 42,097 | | 24,372,837 | 579 | 1,029 | 42,097 | 3,018 | 39,079 |
|  | | C2 | 42,210 | | 24,359,047 | 577 | 1,013 | 42,210 | 3,026 | 39,184 |
|  | | C3 | 43,734 | | 25,639,374 | 586 | 1,056 | 43,734 | 3,177 | 40,557 |
|  | | T1 | 43,924 | | 26,193,583 | 596 | 1,077 | 43,924 | 3,372 | 40,552 |
|  | | T2 | 44,087 | | 26,641,035 | 604 | 1,106 | 44,087 | 3,341 | 40,746 |
|  | | T3 | 42,853 | | 25,879,401 | 604 | 1,103 | 42,853 | 3,194 | 39,659 |
|  | | All | 36,339 | | 33,757,047 | 929 | 1,615 | 36,339 | 5,669 | 30,670 |

N50 indicates the median length of all non-redundant sequences, and the higher N50 value represents the better quality of assembly. Total Consensus Sequences represents all assembled unigenes. Distinct Clusters mean the cluster unigenes. Distinct Singletons mean that this unigene comes from a single gene.

**Supplementary Table 3** Summary of annotation and CDS results

|  | Number of unigene hits | Percentage^a^ |
| --- | --- | --- |
| All-unigenes | 36,339 |  |
| All annotated unigenes | 24,738 | 68.08% |
| Annotated to NR database | 22,681 | 62.42% |
| Annotated to NT database | 20,979 | 57.73% |
| Annotated to Swiss-Prot database | 20,848 | 57.37% |
| Annotated to KEGG database | 16,937 | 46.61% |
| Annotated to COG database | 7,641 | 21.03% |
| Annotated to GO database | 14,767 | 40.64% |
| Total CDS | 23,803 | 65.50% |
| Mapped to the protein database | 22,694 | 62.45% |
| Predicted CDS | 1,109 | 3.05% |

^a^ Proportion of the 36339 assembled All-unigenes

**Supplementary Table 4 Primers used for Q-PCR analysis**

| Gene | Forward primer (5'-3') | Reverse primer (5'-3') | Size |
| --- | --- | --- | --- |
| 6PGD | CTGCTGCTGGACTCCTTCTT | GTTGTGTCTGTAACCGTCGTAA | 144 |
| 18S rRNA | TCATTCCGATAACGAACGAG | GGACATCTAAGGGCATCACA | 143 |
| β-actin | GTGCGTGACATCAAGGAGAAG | CGAGGAAGGATGGCTGGAA | 178 |
| ACC | ACTTCTGCTGTGGTTGTCCTAT | GCATCCATCGTGGGTCATA | 131 |
| ADH | AGTCCGCCTAGATGCTTTGA | GAGCCCACCTCCACCTTAG | 228 |
| AIF1 | ATGCTTGATTGCCACTGGTG | GCAAGTTCACTGCCCAAGAA | 183 |
| AMPK | GAAAGACACACGGCCCAAAC | CTGGCCTGAGTCGAACCTTT | 150 |
| ATGL | ATCTGGTGAAGGTGCTGAAGT | CTCTGACTGTGGCAGGTTGT | 266 |
| C3 | CTGCGAGACTTTACACCACG | CAGCCCGAGTGAGAGAAGAA | 159 |
| CaMKKβ | AAGACGCAACCAGTGGAACT | CACCTCAATCAACGTGCAGC | 195 |
| Caspase 3 | CGCTGCCTGATCATCAACAA | GGATGCGTTTTCACTGTGGT | 198 |
| CIDEC | GAAAAGTGTGAGCTGGACCG | TGCATGGAGAAGAGCATCCA | 197 |
| CPT I | CGCTCCTGCTCCAATGAGA | GAGACCACATAGAGGCAGAAGA | 173 |
| DDIT4 | GACCCTTACCTCGTACCGAC | AATCACCCGAAAACCAGTGC | 156 |
| ELOVL | GGTGGGCAGGAGTCAAGTAT | TACCAGCTGCAGTGAAGTCA | 167 |
| FAS | CATCATCACTGGAGGTCTTGGA | TACGAATGCCTGATCTGGAAGT | 104 |
| G6PC | ATTTAGCCTCGCCTCGTCAG | CCAATCACAGCCACCCAGAT | 117 |
| G6PD | GAGAAGCCTGCCTCAACCA | GGATCGTCCAAGTAGCCAAGT | 158 |
| GAPDH | AAAGTCATCCCCGAGCTCAA | CTTCAGACGCAGCCTTCATC | 148 |
| GS | CTGACCCCATCCTGACCAAC | CAGCCACGCACAAAGTCATC | 142 |
| HK | ACATGGAGGAGCTGCGTAAC | TGTCCAAGGCTCCATCATCT | 102 |
| HMGR | GTGCTGGAGTTGTCACGAGA | GCTGTTCATGGACAGAGGCT | 192 |
| HPRT | CCTCTCCGACTCACAGCTAG | GTCGCCATCTTCACCTCAAC | 136 |
| HSL | ACCATTGCTCCACCGTCTG | CGTCTCACTATCCTGTCCTTCA | 93 |
| ICDH | CATCACAGGCTTCGCTCAC | CTCTGGCTTGTAATGCTTCTCA | 151 |
| ILF2 | CATGCTGACCAATGAGACGG | CACCGTAGACTGAGAGGCAT | 193 |
| LepR | CCTCAAAACAGCCAAGCACC | CGCTCTGAGGCTGGTATCTG | 166 |
| LKB1 | TTGGTCTGCGGGAGTAACAC | GACAGCAGAGGTCCACACTC | 135 |
| LPL | AGTCCGATCAACACGAAGC | GGTGCCGTTCCCATTTAG | 208 |
| ME | TGAGGCATCGCAGAGTGAG | AGCAGCAGTTCCTTGAATGTC | 194 |
| NAMPT | ATTACGGCACTAAGGACCCC | AGGTCCTCTCCCCAGATCTT | 196 |
| PEPCK | TGTGGATATGGGTGCGCTTT | TCCAACTGCCTCAACTCGTC | 135 |
| PFK2 | TCTCCAAGAAACTCACCCGC | CCTGCGTATCTTCATGGCCT | 149 |
| PYG | GTACGGCGTGAAGATCAACC | CGATGATCACAGTCCGAGGA | 161 |
| RPL7 | GCGCCAGATCTTCAATGGAG | CTCATTCTGCCATGACCACG | 147 |
| SOCS2 | GGACAGCTCACAGAGGGATT | AGCTGCACTGTTCCATTTGG | 246 |
| TAK1 | CCAACCAGGGGAGCAAGAAT | TCGAGTTTGGACATGGAGCC | 195 |
| TAP2 | GCAGTCAGTGAGCGGTATTG | GTCTTCTTGCCAGCACCAAA | 194 |
| TBP | AGTCCCATGATGCCCTATGG | GCAACAGCTTGGGAATGGAA | 179 |
| TUBA | CACTTCCCTCTTGCCACCTA | ACGGTACAGGAGACAACAGG | 165 |
| UBCE | GCCCGTGGAAGGATTCAAAA | AAGGCAGGTGGAGAGTATGG | 156 |
| Ubiquilin | GCAAGGCTTCATGGGAGATG | AAGTGGTGGAGTCCTTCAGG | 155 |

**Supplementary Table 5** DEGs between control and Zn treatment group from transcriptome analysis.

| DEGs | KEGG | Up-Down-Regulation | Log_2_(Ratio) | Pathway |
| --- | --- | --- | --- | --- |
| NADH-Q | K03880 | Up | 1.1 | [Oxidative](javascript:void(0);) [phosphorylation](javascript:void(0);) |
| SDH | K00235 | Up | 0.8 | [Oxidative](javascript:void(0);) [phosphorylation](javascript:void(0);) |
| Cyt C Ox | K02256 | Up | 0.5 | [Oxidative](javascript:void(0);) [phosphorylation](javascript:void(0);) |
| ATP synthase (F-type subunit alpha) | K02132 | Up | 0.5 | [Oxidative](javascript:void(0);) [phosphorylation](javascript:void(0);) |
| ATP synthase (V-type proteolipid subunit) | K02155 | Up | 1.5 | [Oxidative](javascript:void(0);) [phosphorylation](javascript:void(0);) |
| ATP synthase (V-type  S1 subunit) | K03662 | Up | 0.5 | [Oxidative](javascript:void(0);) [phosphorylation](javascript:void(0);) |
| PARP | K15259 | Up | 6.6 | Apoptosis |
| AIF | K04727 | Up | 1.6 | Apoptosis |
| DFFA | K02310 | Up | 1.1 | Apoptosis |
| DFFB | K02311 | Up | 1.1 | Apoptosis |
| CASP3 | K02187 | Up | 2.5 | Apoptosis |
| NF-κB | K04735 | Up | 1.0 | Apoptosis |
| Calpain | K01367 | Up | 1.7 | Apoptosis |
| Chemokine 7 | K05509 | Up | 0.7 | Chemokine signaling pathway |
| Chemokine 2 | K14624 | Up | 5.4 | Chemokine signaling pathway |
| NFAT | K04446 | Up | 1.0 | B cell receptor signaling pathway |
| CRT | K08057 | Up | 0.8 | Endoplasmic Reticulum Stress |
| PERK | K08860 | Up | 0.6 | Endoplasmic Reticulum Stress |

**Supplementary Fig. 1** (A) Length distribution of All-Unigenes. (B) Length distributions of blasted CDS nucleotide sequence. All-Unigene.blast.cds.fa represents the CDS which mapped to the protein database. (C) Length distributions of predicted CDS nucleotide sequence. All-Unigene.ESTscan.cds.fa represents the predicted CDS by ESTscan.


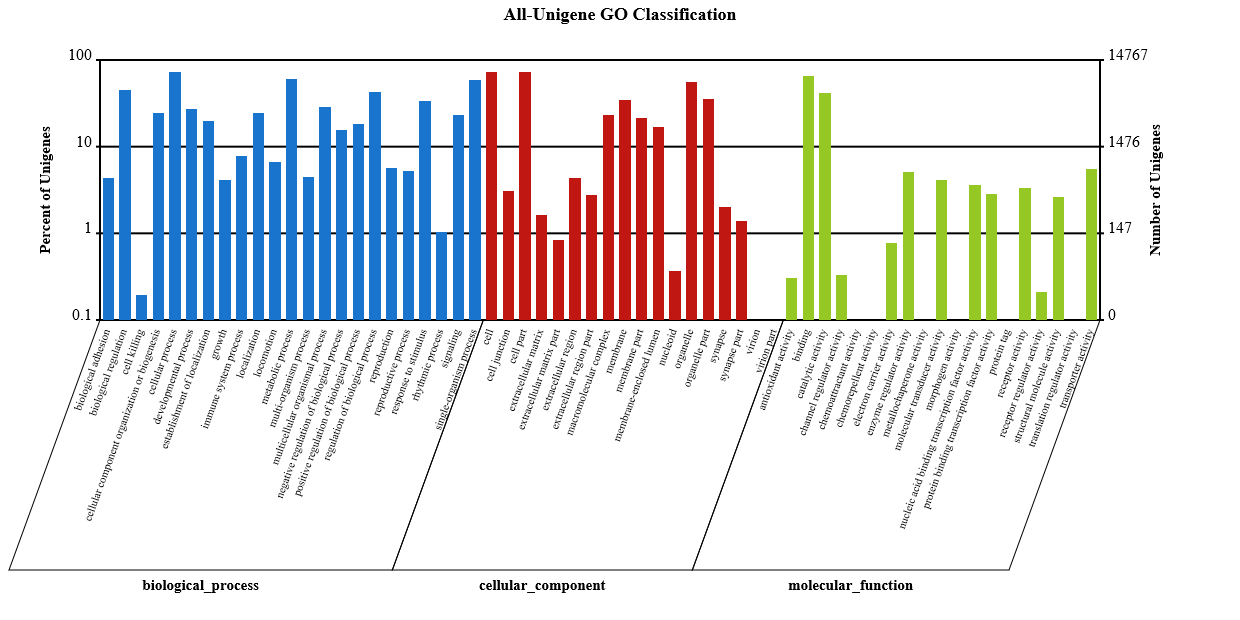


**Supplementary Fig. 2** GO classification analysis of Unigenes in All-Unigene. GO functions is showed in X-axis. The right Y-axis shows the number of genes which have the GO function. The left Y-axis shows the percentage.


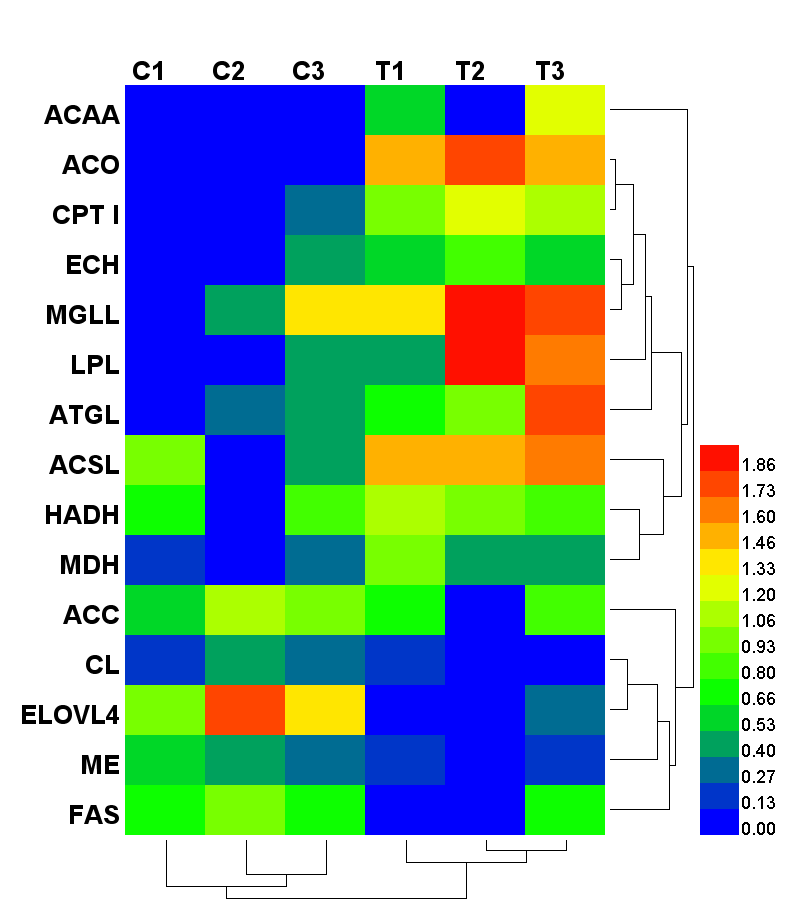


**Supplementary Fig. 3** Heatmap of DEGs in lipid metabolism of *S. hasta* (control: C1, C2 and C3; Zn exposed groups: T1, T2, T3). The heatmap was generated by Heatmap Illustrator, version 1.0. The gene expression levels in the heatmap were shown from low (blue), middle (green) to high (red). The full names of the genes were provided in abbreviation list of supplementary file.


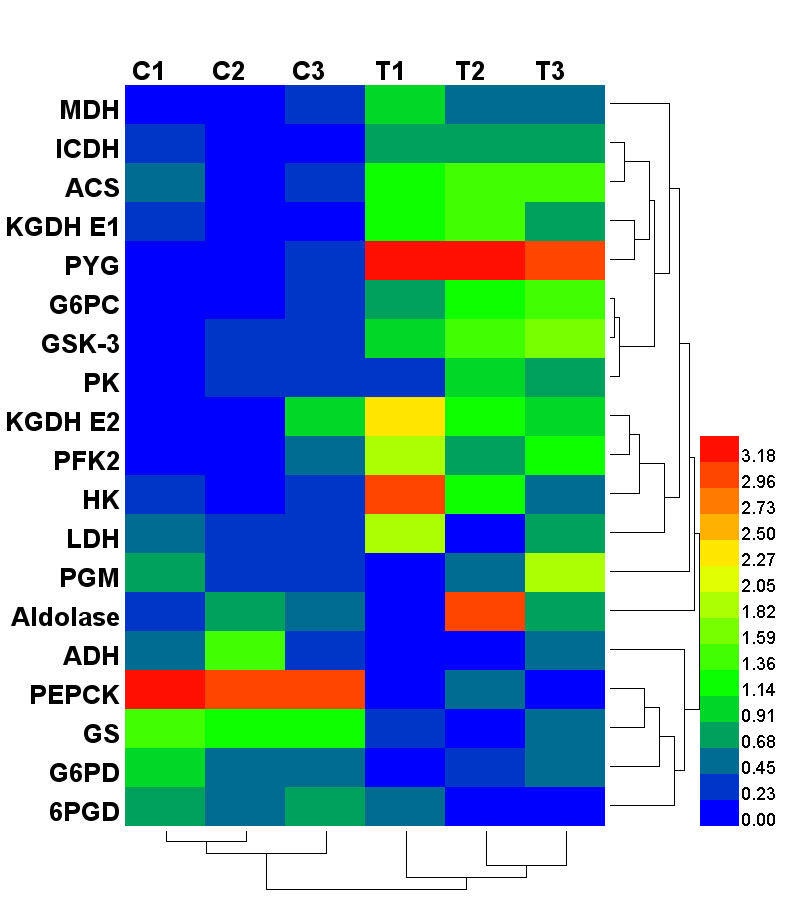


**Supplementary Fig. 4** Heatmap of DEGs in carbohydrate metabolism of *S. hasta* (control: C1, C2 and C3; Zn-exposed group: T1, T2 and T3). The heatmap was generated by Heatmap Illustrator, version 1.0. The gene expression levels in the heatmap were shown from low (blue), middle (green) to high (red). The full names of the genes were provided in abbreviation list of supplementary file.


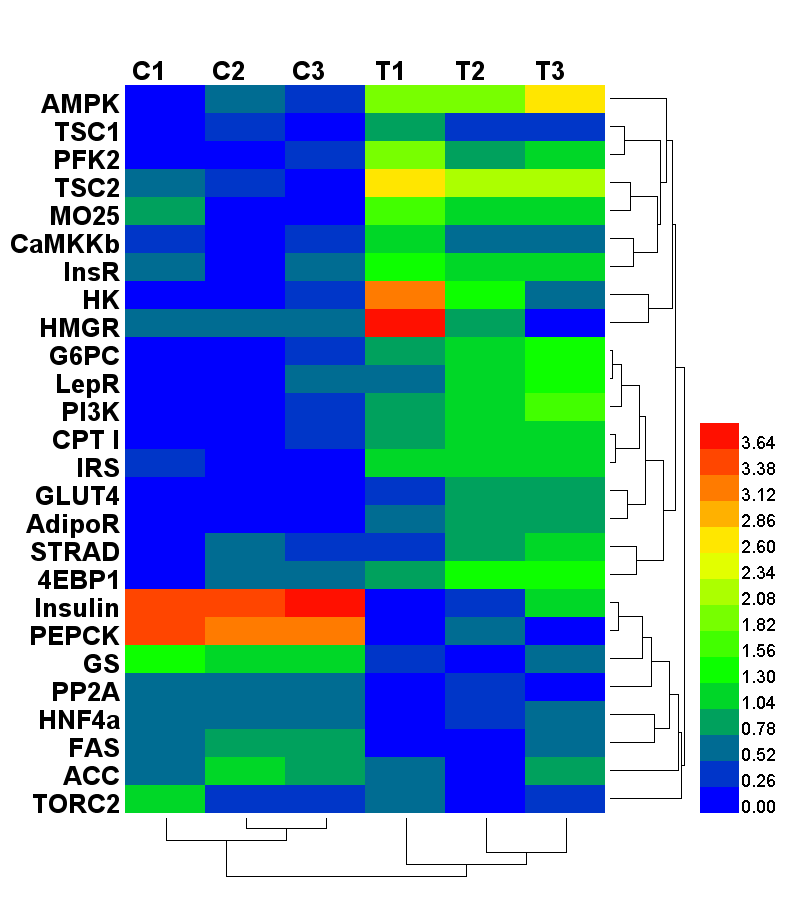


**Supplementary Fig. 5** Heatmap of DEGs in AMPK signaling pathway of *S. hasta* (control: C1, C2 and C3; Zn-exposed group: T1, T2 and T3). The heatmap was generated by Heatmap Illustrator, version 1.0. The gene expression levels in the heatmap were shown from low (blue), middle (green) to high (red). The full names of the genes were provided in abbreviation list of supplementary file.


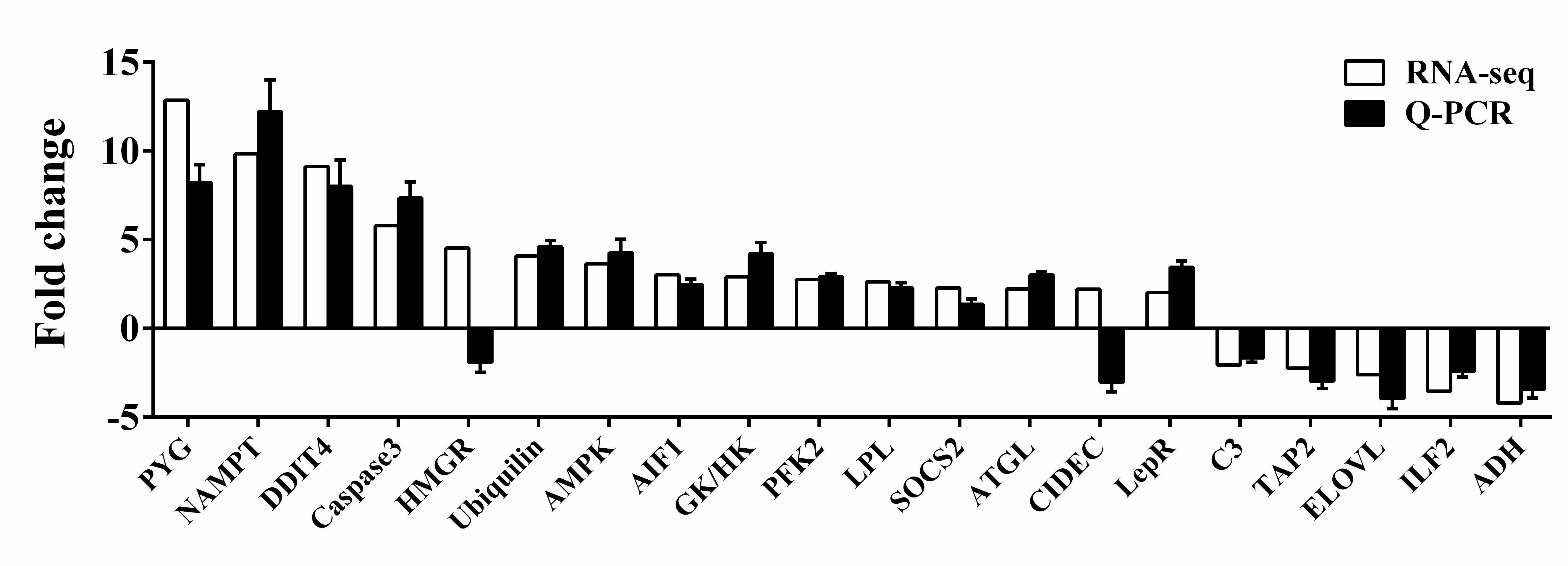


**Supplementary Fig. 6** Comparison of mRNA levels between RNA-seq and Q-PCR results. The y-axis is the fold change of genes’ mRNA expression and the x-axis is the gene name.

**
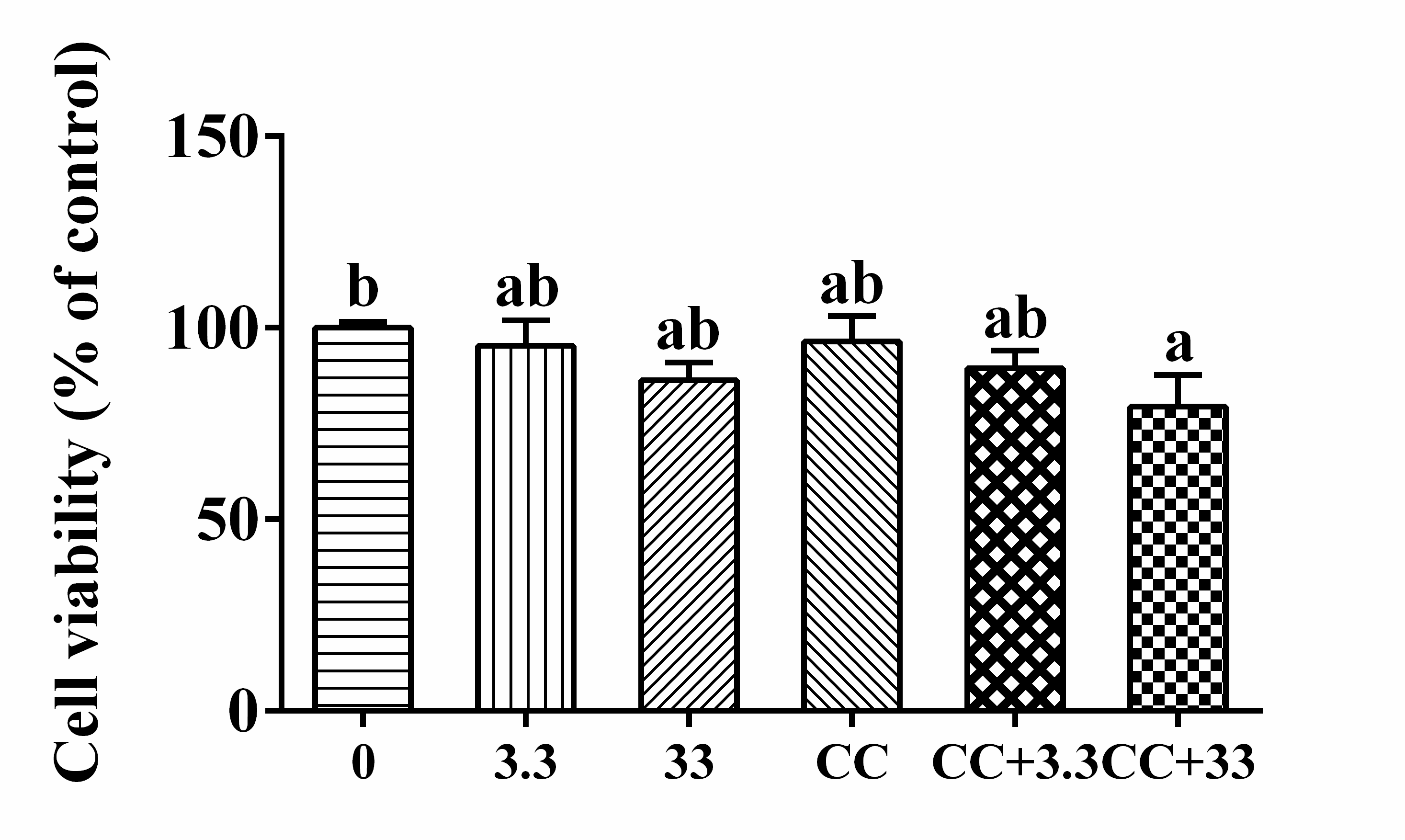
**

**Supplementary Fig. 7** Effect of Zn and/or Compound C (CC) on cell viability in primary hepatocytes from *S. hasta*. (0: control; 3.3: 3.3 µM Zn; 33: 33µM Zn; CC: 200 nM Compound C; CC + 3.3: 200 nM CC + 3.3 µM Zn; CC + 33: 200 nM CC + 33 µM Zn). Values are mean ± SEM (n = 4). Bars that share different letters indicate significant differences among groups (p < 0.05).
